# 529. Safety of AZD7442 (Tixagevimab/Cilgavimab) for Treatment of Mild-to-Moderate COVID-19: 15-Month Final Analysis of the TACKLE Phase 3 Study

**DOI:** 10.1093/ofid/ofad500.598

**Published:** 2023-11-27

**Authors:** F D Richard Hobbs, Hugh Montgomery, Francisco Padilla, Jesus Abraham Simón Campos, Douglas Arbetter, Seth Seegobin, Alexandre Kiazand, Katie Streicher, Nuria Martinez-Alier, Taylor Cohen, Mark T Esser

**Affiliations:** University of Oxford, Oxford, England, United Kingdom; University College London, London, England, United Kingdom; Centro de Investigación en Cardiología y Metabolismo, Guadalajara, Jalisco, Mexico; Köhler & Milstein Research/Hospital Agustín O’Horán, Mérida, Yucatan, Mexico; AstraZeneca, Waltham, Massachusetts; AstraZeneca, Waltham, Massachusetts; AstraZeneca, Waltham, Massachusetts; AstraZeneca, Waltham, Massachusetts; AstraZeneca, Waltham, Massachusetts; AstraZeneca, Waltham, Massachusetts; AstraZeneca, Waltham, Massachusetts

## Abstract

**Background:**

In the TACKLE phase 3 outpatient treatment study, 600-mg AZD7442 (tixagevimab/cilgavimab) in adults with mild to moderate COVID-19 significantly reduced progression to severe disease or death over 29 days and was well-tolerated at primary analysis. Here, we report final safety findings from TACKLE.

**Methods:**

In TACKLE (NCT04723394), non-hospitalized adults with mild to moderate COVID-19 were randomized 1:1 and dosed ≤7 days from symptom onset with 600-mg AZD7442 (N=452) or placebo (N=451). Results are reported from the January 22, 2023 final data cut-off. The primary safety endpoint was assessment of adverse events (AEs), serious adverse events (SAEs), and AEs of special interest (AESIs). A cardiovascular events adjudication committee independently evaluated 5 event types (cardiac ischemia, cardiovascular death, heart failure, stroke, and thrombotic events).

**Results:**

Across both the AZD7442 and placebo groups, 797 (87.6%) participants completed the study. Median follow-up was ∼15 months or 458.5 days in the AZD7442 group and 458.0 days in the placebo group. AEs occurred in 251 (55.5%) and 252 (55.9%) of participants administered AZD7442 and placebo, respectively (**Table**). The most common AEs were COVID-19, post-acute COVID-19 syndrome, and COVID-19 pneumonia. Reinfection with COVID-19 within 6 months occurred in 1 (0.2%) and 2 (0.4%) participants in the AZD7442 and placebo groups, respectively (all other COVID-19 AEs were judged to be sequelae from the original event). Most AEs were mild to moderate in severity; 33 (7.3%) and 51 (11.3%) of participants in the AZD7442 and placebo groups, respectively, reported an AE of grade 3 (severe) or 4 (life-threatening). SAEs occurred in 46 (10.2%) and 65 (14.4%) AZD7442 and placebo participants, and deaths in 8 (1.8%) and 8 (1.8%), respectively. AESIs occurred 4.2% and 3.8% of AZD7442 and placebo participants, respectively, including 0.4% and 0.2% with cardiovascular disorders categorized as AESIs. Cardiovascular events occurring in 4 (0.9%) participants in both groups were evaluated by an adjudication committee, with 1 (0.2%) participant in both groups having a positively adjudicated event.

**Figure:**
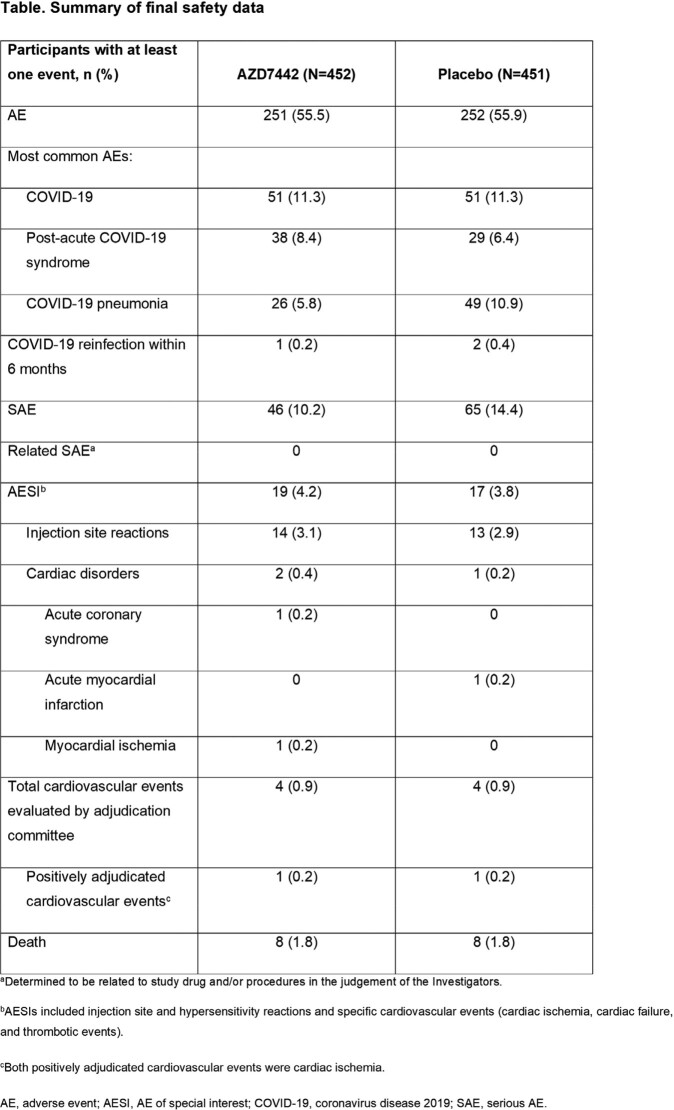

**Conclusion:**

This analysis provides further evidence of the long-term safety of AZD7442 as treatment for COVID-19.

**Disclosures:**

**F.D. Richard Hobbs, FMedSci**, AstraZeneca: Grant/Research Support|National Institute for Health and Care Research: Grant/Research Support|UK Research and Innovation: Grant/Research Support **Hugh Montgomery, MD**, AstraZeneca: Advisor/Consultant|Millfield Medical Electronics Ltd: Advisor/Consultant **Francisco Padilla, MD**, Amgen: Grant/Research Support|AstraZeneca: Grant/Research Support|Boehringer Ingelheim: Grant/Research Support|Ferrer: Grant/Research Support|Kowa: Grant/Research Support|Medix: Grant/Research Support|Merck: Grant/Research Support|Merck Sharp and Dohme: Grant/Research Support|Novartis: Grant/Research Support|Pfizer: Grant/Research Support|Sanofi: Grant/Research Support|Servier: Grant/Research Support|Silanes: Grant/Research Support **Jesus Abraham Simón Campos, MD**, AstraZeneca: Expert Testimony|Atea: Advisor/Consultant|Eli Lilly: Advisor/Consultant|Pfizer: Expert Testimony|Regeneron: Expert Testimony|Roche: Expert Testimony **Douglas Arbetter, MPH**, AstraZeneca: Employement|AstraZeneca: Stocks/Bonds **Seth Seegobin, PhD**, AstraZeneca: Employee|AstraZeneca: Stocks/Bonds **Alexandre Kiazand, MD**, AstraZeneca: Employee|AstraZeneca: Stocks/Bonds **Katie Streicher, PhD**, AstraZeneca: Employee|AstraZeneca: Stocks/Bonds **Nuria Martinez-Alier, PhD**, AstraZeneca: Employee|AstraZeneca: Employement|AstraZeneca: Stocks/Bonds|AstraZeneca: Stocks/Bonds **Taylor Cohen, PhD**, AstraZeneca: Employement|AstraZeneca: Stocks/Bonds **Mark T. Esser, PhD**, AstraZeneca: Employee|AstraZeneca: Stocks/Bonds

